# The Optimal Outcome of Suppressing Ewing Sarcoma Growth *in vivo* With Biocompatible Bioengineered miR-34a-5p Prodrug

**DOI:** 10.3389/fonc.2020.00222

**Published:** 2020-02-25

**Authors:** Dai-Feng Li, Ying Yuan, Mei-Juan Tu, Xiang Hu, Yi-Zhou Li, Wan-Rong Yi, Peng-Cheng Li, Yong Zhao, Zhen Cheng, Ai-Ming Yu, Chao Jian, Ai-Xi Yu

**Affiliations:** ^1^Department of Orthopedics Trauma and Microsurgery, Zhongnan Hospital of Wuhan University, Wuhan, China; ^2^Molecular Imaging Program at Stanford (MIPS), Bio-X Program, Department of Radiology, Canary Center at Stanford for Cancer Early Detection, Stanford University, Stanford, CA, United States; ^3^Department of Biochemistry & Molecular Medicine, UC Davis School of Medicine, Sacramento, CA, United States

**Keywords:** RNA therapy, miR-34a, bioengineered, apoptosis, Ewing Sarcoma, mouse model

## Abstract

Being the second most common type of primary bone malignancy in children and adolescents, Ewing Sarcoma (ES) encounters the dilemma of low survival rate with a lack of effective treatments. As an emerging approach to combat cancer, RNA therapeutics may expand the range of druggable targets. Since the genome-derived oncolytic microRNA-34a (miR-34a) is down-regulated in ES, restoration of miR-34a-5p expression or function represents a new therapeutic strategy which is, however, limited to the use of chemically-engineered miRNA mimics. Very recently we have developed a novel bioengineering technology using a stable non-coding RNA carrier (nCAR) to achieve high-yield production of biocompatible miRNA prodrugs, which is a great addition to current tools for the assessment of RNA therapeutics. Herein, for the first time, we investigated the biochemical pharmacology of bioengineered miR-34a-5p prodrug (nCAR/miR-34a-5p) in the control of ES using human ES cells and xenograft mouse models. The bioengineered nCAR/miR-34a-5p was precisely processed to mature miR-34a-5p in ES cells and subsequently suppressed cell proliferation, attributable to the enhancement of apoptosis and induction of G2 cell cycle arrest through downregulation of SIRT-1, BCL-2 and CDK6 protein levels. Furthermore, systemic administration of nCAR/miR-34a-5p dramatically suppressed the ES xenograft tumor growth *in vivo* while showing biocompatibility. In addition, the antitumor effect of bioengineered nCAR/miR-34a-5p was associated with a lower degree of tumoral cell proliferation and greater extent of apoptosis. These findings demonstrate the efficacy of bioengineered miR-34a-5p prodrug for the treatment of ES and support the development of miRNA therapeutics using biocompatible bioengineered miRNA prodrugs.

## Introduction

MicroRNAs (miRNAs or miRs) are a superfamily of single-stranded noncoding RNAs (ncRNAs) consisting of 18-25 nucleotides that are derived from the genome (1–3). Through post-transcriptional regulation of target gene expression, miRNAs may control particular cellular pathways and play an important role in disease initiation, progression, and prognosis (4–8). Many miRNAs have been revealed to be specifically dysregulated in human carcinoma cells, among which some downregulated miRNAs (e.g., miR-34a-5p) function as tumor suppressors and upregulated miRNAs (e.g., miR-21-5p) exhibit tumor promotive activities (9–11). With the improved understanding of miRNAs in cancer biology, miRNA-based therapeutic strategies are emerging, to restore tumor suppressive miRNAs lost in carcinoma cells or inhibit tumor promotive miRNAs overexpressed in tumor, which may expand the range of druggable targets and represent new ways for the treatment of cancer disease (12–15).

As the second most frequent primary malignant bone tumor, Ewing Sarcoma (ES) has a predilection in children and young adults (16, 17). Genetically, ES is characterized by balanced chromosomal translocations and fusions of the FET gene family with an ETS transcription factor, of which Ewing Sarcoma breakpoint region 1 protein- Friend leukemia integration 1 transcription factor (EWSR1-FLI1) fusion accounts for 85% (18, 19). Other rare mutations include TP53, STAG2, and CDKN2A deletions (20, 21). Moreover, ES is generally locally aggressive and has high metastatic potential, resulting in a 5-year survival rate lower than 30% (22–25). Currently, surgery, radiotherapy and chemotherapy have been introduced to offer effective remedy against ES. However, the past several decades has witnessed negligible progression in prolonging patients' long-term survival rate (25–27). Therefore, there is an urgent demand to develop novel therapeutic strategies to combat aggressive or recurrent ES that would help improve the treatment of ES.

Among various cancer-related miRNAs, microRNA-34a (miR-34a) is downregulated in varied types of cancers (28–31). There are also increasing evidences showing that miR-34a is significantly down-regulated in ES (32, 33) and a lower miR-34a expression level is correlated with a notably poor prognosis in ES patients (34). As a well-defined master tumor suppressor under p53-governed transcriptional regulation, miR-34a controls cell proliferation and apoptosis, cell cycle, senescence, and invasion through regulation of a variety of targets, such as silent information regulator-1 (SIRT-1) (35, 36), cyclin-dependent kinase 6 (CDK6) (37), B-cell lymphoma-2 (BCL-2) (38, 39), hepatocyte growth factor receptor (c-MET) (30), and cell surface glycoprotein (CD44) (40). Moreover, it has been demonstrated that reintroduction of miR-34a is effective in suppressing osteosarcoma (38), liver cancer (30), prostate cancer (40), and colorectal cancer (31). However, to date, the efficacy of miR-34a for the treatment of ES has not been determined, either *in vitro* or *in vivo*. In this regard, we intended to assess the effectiveness of exogenously introduced miR-34a for the control of ES and delineate the underlying pharmacological actions.

Currently, RNA therapeutics mainly rely on chemically-engineered RNA agents or their corresponding DNA materials. In spite of easy accessibility of chemically-engineered RNA mimics, there are concerns about the unknown alterations in higher-order structures, activities and biosafety profiles caused by excessive artificial modifications (41, 42). In the past several years, large efforts have been made to produce bioengineered or biologic ncRNA agents in living cells (41–43). Very recently, our group have developed a novel ncRNA bioengineering technology using a stable tRNA/pre-miRNA-based ncRNA carrier (nCAR) to achieve high-yield and large-scale production of biocompatible miRNA prodrugs (43, 44). Distinguished from chemically-engineered miRNA mimics, bioengineered miRNA prodrugs are produced and folded within living cells while carrying no or minimal necessary posttranscriptional modifications (44–47). Our studies have demonstrated that high level of mature miR-34a-5p is selectively released from novel bioengineered miR-34a-5p prodrug to control target gene expression, and is effective to inhibit human lung cancer cell proliferation and xenograft tumor growth (44). Further studies have also demonstrated that bioengineered miR-1291-5p prodrug is able to exhibit potent anticancer efficacy both in monotherapy and in combination treatment with chemotherapeutics (46). These findings suggest that bioengineered miRNA agents represent a new class of ncRNA molecules for research on miRNA biology and assessment of miRNA therapeutics.

**Scheme 1:**
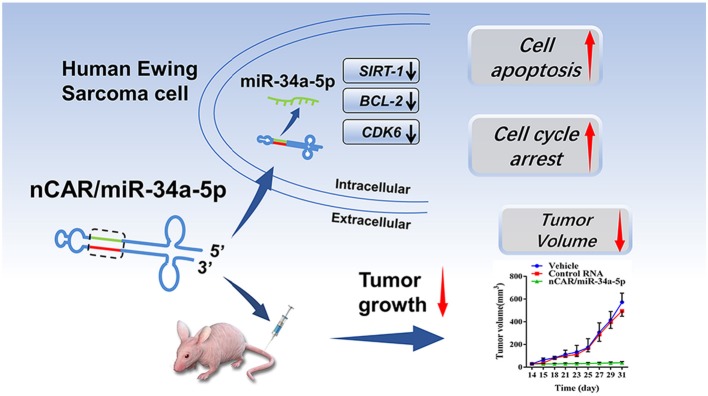
High levels of mature miR-34a-5p was selectively released from bioengineered nCAR/miR-34a-5p to control Ewing Sarcoma through the downregulation of target oncogene expression and induction of apoptosis.

Herein, we investigated the biochemical pharmacology of our novel bioengineered miR-34a-5p prodrug (nCAR/miR-34a-5p) in the control of ES both *in vitro* and *vivo*. Our *in vitro* data demonstrated that bioengineered nCAR/miR-34a-5p was precisely processed to target warhead miR-34a-5p and subsequently, suppressed ES cell proliferation. The antiproliferative activity of biologic miR-34a-5p prodrug was associated with the enhancement of apoptosis and the induction of G2 cell cycle arrest through downregulation of SIRT-1, BCL-2 and CDK6 protein levels. Furthermore, systemic administration of nCAR/miR-34a-5p dramatically suppressed the ES xenograft tumor growth *in vivo* while well tolerated in mice (shown in Scheme 1). The antitumor effect of biologic miR-34a-5p prodrug was linked to a lower degree of tumoral cell proliferation and greater extent of apoptosis. These findings demonstrated the antitumor activity of bioengineered miR-34a-5p prodrug and supported the development of miRNA therapeutics using novel biocompatible bioengineered miRNA agents.

## Materials and Methods

### Reagents

Trizol reagent, BCA Protein assay kit, 0.05% trypsin-EDTA, and RIPA lysis buffer were purchased from Thermo Fisher Scientific (Waltham, MA). Protease inhibitor cocktail was purchased from Roche Diagnostics (Mannheim, Germany). Lipofectamine™ 3000 transfection reagent was bought from Invitrogen (Carlsbad, USA). Polyvinylidene fluoride (PVDF) membrane, western blot ECL substrate kit, and blotting-grade blocker were purchased from Bio-Rad (Hercules, CA, USA). *In vivo*-jetPEI^TM^ was purchased from Polyplus-transfection (NY, USA). Alanine aminotransferase (ALT) assay kit (C009-2-1), aspartate aminotransferase (AST) assay kit (C010-2-1), blood urea nitrogen (BUN) assay kit (C013-2-1), creatinine (Cr) assay kit (sarcosine oxidase) (C011-2-1), total bilirubin (TB) assay kit (C019-1-1) and cardiac troponin-I (cTnI) assay kit (H149-1) were all purchased from Nanjing Jiancheng Bioengineeing Institute, Nanjing, China. All other solvents and chemicals of analytical grade were purchased from either Thermo Fisher Scientific or Sigma-Aldrich (St. Louis, MO).

### Human Ewing Sarcoma Cell Line

The human ES cell line A673 was purchased from the American Type Culture Collection (ATCC, Manassas, VA) and the human ES cell line RD-ES was a gift from the Musculoskeletal Tumor Center, Peking University People's Hospital (M.S.T.C., PKUPH). Cells were cultured in Dulbecco's modified Eaglemedium (DMEM) containing high glucose (Gibco), supplemented with 10% fetal bovine serum (Gibco) at 37°C in a humidified environment containing 5% carbon dioxide. The cells were expanded in tissue culture dishes and passaged or harvested when reaching 80–90% confluency. For *in vitro* cellular studies, cells were treated in triplicate and assayed separately.

### Production of Bioengineered nCAR/miR-34a-5p

Sequences of miRNAs and pre-miR-34a were obtained from miRBase (http://www.mirbase.org/) for a construction of ncRNA expression plasmids, which were then transformed into HST08 *E. coli* followed by fermentation. Anion exchange fast protein liquid chromatograph (FPLC) purification was conducted as previously described (43, 44). Separation of nCAR/miR-34a-5p from total RNAs was achieved on an Enrich-Q 10 × 100 column. The purity of the bioengineered RNA agents was determined quantitatively by high-performance liquid chromatography (HPLC) analysis. Highly purified (over 98% by HPLC) recombinant non-coding RNAs including nCAR/miR-34a-5p and control RNA were used in this study. Lipofectamine 3000 and *in vivo*-jetPEI were employed to deliver RNA molecules into cells and animals, respectively.

### Reverse Transcription Quantitative Real-Time PCR (RT-qPCR)

Human ES A673 cells were seeded in 6-well plate (5 × 10^5^ cells/well) and transfected with 15 nM nCAR/miR-34a-5p, control RNA, or vehicle for 48 h. Cells were harvested with TRIpure Total RNA Extraction kit (EP013; ELK Biotechnology, Wuhan, China). Reverse transcription was performed using M-MLV Reverse Transcriptase (EQ002; ELK Biotechnology, Wuhan, China) and random hexamers (for U6) or stem-loop primer 5′-CTC AAC TGG TGT CGT GGA GTC GGC AAT TCA GTT GAG ACA ACC AG-3′ (for miR-34a-5p). Then qPCR analyses were performed with QuFast SYBR Green PCR Master Mix (EQ001; ELK Biotechnology, Wuhan, China) and the following gene specific primers (44), forward 5′-AGG CAG TGT CTT AGC TGG TTG T-3′, reverse 5′-CTC AAC TGG TGT CGT GGA GTC-3′ for miR-34a-5p, and forward 5′-CTC GCT TCG GCA GCA CAT-3′, reverse 5′-AAC GCT TCA CGA ATT TGC GT-3′ for U6, on a StepOne™ Real-Time PCR system (Life technologies, Carlsbad, CA, USA). Relative mature miR-34a-5p levels were calculated by using the comparative threshold cycle (Ct) method with the formula 2^−ΔΔ*Ct*^.

### Protein Isolation and Western Blot Analyses

Human ES A673 cells were seeded in 6-well plates (5 × 10^5^ cells/well) and transfected with 15 nM nCAR/miR-34a-5p, control RNA, or vehicle for 48 h. Total proteins of the harvested cells or tumor tissues were isolated with RIPA lysis buffer supplemented with complete protease inhibitors. Protein concentrations were quantitated using a BCA Protein Assay Kit. Proteins (30 μg/lane) were separated on a 10% SDS-PAGE gel and electrophoretically transferred onto PVDF membranes. After a 2-h incubation with 5% nonfat milk, membranes were incubated with anti-SIRT-1 (1:1000; AB196495; Abcam, Cambridge, UK), anti-BCL-2 (1:1000; AB196495; Abcam, Cambridge, UK), anti-CDK6 (1:1000; AB151247; Abcam, Cambridge, UK), anti-p53 (1:2000; #2524; Cell Signaling Technology, MA, USA) or anti-GAPDH (1:10000; AB37168; Abcam, Cambridge, UK) rabbit antibody, and then with a peroxidase goat anti-rabbit IgG (1:10000; AS1107; Aspen, Wuhan, China). After incubation with Clarity Western ECL substrates (AS1059; Aspen, Wuhan, China), the blots were visualized with the imager (LiDE110; Canon) and quantified by Alpha Ease FC software (Alpha Innotech, Silicon Valley, CA, USA). GAPDH was used as a loading control.

### CCK-8 Assay

Human ES A673 (5 × 10^3^ cells/well) and RD-ES (5 × 10^3^ cells/well) were, respectively seeded in 96-well plates and cultured overnight. Then cells were transfected with bioengineered nCAR/miR-34a-5p or control RNA (0, 1, 2, 5, 10, 20, 50 nM) using Lipofectamine 3000. The CCK8 method was employed to measure cell viability at 48 h post-treatment, based on previous reports (38, 39). To estimate the pharmacodynamic parameters, cell viability data were fitted to an inhibitory, normalized response model with variable slop, Y = Emin + (Emax−Emin)/[1+10^(*LogEC*50−*X*)^*HillSlope] (GraphPad Prism, San Diego, CA, USA).

### Flow Cytometry Analyses

Human ES A673 cells were seeded in 6-well plates (5 × 10^5^ cells/well) and transfected with either 15 nM nCAR/miR-34a-5p, control RNA, or vehicle for 48 h. For cell apoptosis analyses, cells were incubated with Annexin V-FITC conjugate (5 μl), Annexin V binding buffer (400 μl) in a dark room for 15 min. For cell cycle analyses, cells were fixed in 70% cold alcohol for 1 h and stained with 0.5 ml PI/RNase for 30 min. Samples were analyzed on a Cytomics FC500 Flow Cytometry Analyzer (Beckman Coulter). The rate of increase is calculated following the formula: rate of increase = (apoptotic rate_miRNA_ − apoptotic rate_controlRNA_)/apoptotic rate_controlRNA_.

### Immunofluorescence Analysis of Cell Apoptosis Marker

Human ES A673 cells were seeded onto sterile coverslips (10212432C; Citotest Labware Manufacturing Co., Ltd, Jiangsu, China), which were inserted in 6-well plates (5 × 10^5^ cells/well). 48 h after the transfection with 15 nM nCAR/miR-34a-5p, control RNA or vehicle, the coverslips were fixed with 4% paraformaldehyde (AS1018; Aspen, Wuhan, China). After being permeabilized and blocked, the coverslips were incubated with primary antibody against cleaved-caspase-3 (1:200; AF7022; Affbiotech, OH, USA) at 4°C overnight, and then incubated with FITC labeled goat anti-rabbit secondary antibody (1:50; AS-1110; Aspen, Wuhan, China). Nucleus was counterstained with DAPI (AS1075; Aspen, Wuhan, China). Images were taken and observed with a fluorescence microscope (IX51; Olympus Corporation, Shinjuku, Tokyo, Japan). Cleaved-caspase-3-positive cells were counted under the fluorescence microscope for percentages of positive stained cells which were compared between different groups of treatments (38, 39).

### Animal Studies

The animal protocol in this study was approved by the Institutional Committee on the Ethics of Animal Experiments of Wuhan University, Wuhan, China. All animal procedures were conducted in compliance with the Guide for the Care and Use of Laboratory Animals of the National Institutes of Health. Nude mice (6–8 weeks old, female, 18-22 g) were purchased from Beijing Weitong Lihua Experimental Animals. All mice were monitored closely and euthanized if reached to any one of relevant criteria (tumor size > 2,000 mm^3^ or >2 cm in any direction).

Nude mice were anesthetized via intraperitoneal (i.p.) injection with 1% pentobarbital sodium (35 mg/kg) and human ES A673 cells (3 × 10^6^ cells in 100 μl PBS) were injected subcutaneously into the lateral side of left gluteus maximus. Tumor size was assessed using the following formula: tumor volume (mm^3^) = length × width^2^ × 0.52. Two weeks post-inoculation, mice were assigned into three groups according to tumor sizes and treated with *in vivo*-jetPEI-formulated nCAR/miR-34a-5p, control RNA or vehicle (40 μg/mouse, QOD for 8 times) through tail vein injection. All mice were sacrificed at the end of the study. Tumor tissues and major organs (heart, liver, spleen, lung, and kidney) were dissected, weighted, and fixed in 4% paraformaldehyde for histological analyses and further immunohistochemistry analyses, or in liquid nitrogen for western blot analyses on p53 expression. Blood samples were collected for evaluating ALT, AST, TB, BUN, Cr, and cTnI levels under the microplate reader (DR-200Bs; Diatek, Wuxi, China) following the instructions of animal serum biochemical kits.

### Histological and Immunohistochemistry Analysis

Harvested organs were embedded by optimal cutting temperature (OCT) compound (Tissue-Tek®, Sakura Finetek, USA), cut into 6 μm sections in the cryostat at −20°C with a microtome and subjected to hematoxylin and eosin (H&E) using Shandon™ rapid Chrome kit (Thermo Scientific, USA). Subsequently, the samples were imaged using a MicroPublisher system (Q-Imaging) and analyzed. The expression of Ki-67 and cleaved-caspase-3 were determined by immunohistochemical (IHC) study using primary antibody against Ki-67 (1:400; 27309-1-AP; Proteintech Group, Inc, Wuhan, China), cleaved-caspase-3 (1:100; AF7022; Affbiotech, OH, USA) and the HRP-goat anti-rabbit secondary antibody (1:50; AS-1110; Aspen, Wuhan, China). Images were obtained using the microscope (IX51; Olympus Corporation, Shinjuku, Tokyo, Japan). Image-Pro Plus software (Media Cybernetics Corporation, Rockville, Maryland, US) was used for analyzing the proliferation and apoptosis rate of tumor tissues. The mean density was used to evaluate the content of Ki-67 or cleaved-caspase-3 following the formula: mean density = integrated option density (IOD)/total area of target tissue (38, 39).

### Statistical Analyses

All data were analyzed with Graphpad Prism 6 (GraphPad Software, San Diego, CA, USA). Statistical analyses were conducted by using one-way or two-way analysis of variance (ANOVA). Differences were considered as statistically significant at the level of *P*-value < 0.05.

## Results

### Bioengineered nCAR/miR-34a-5p Is Processed to Mature miR-34a-5p to Control Target Gene Expression and Inhibit Ewing Sarcoma Cell Proliferation

**Figure 1:**
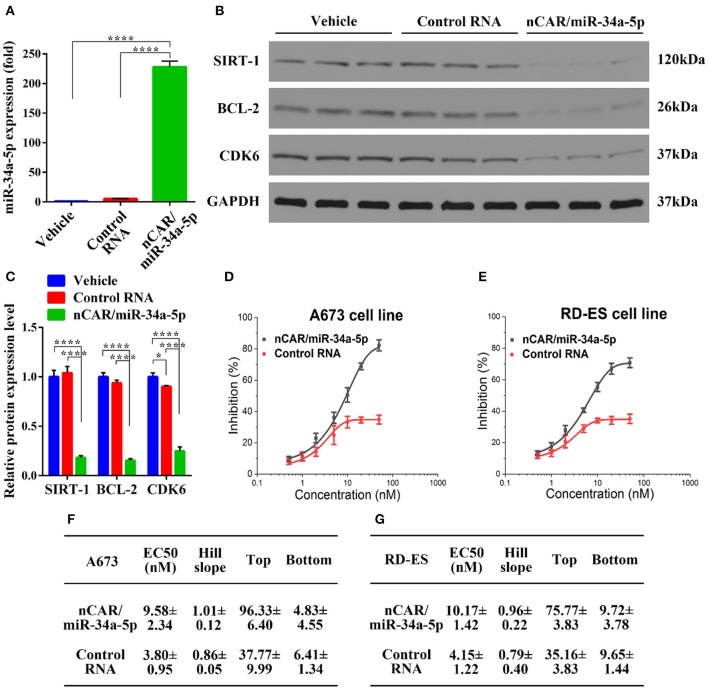
Bioengineered nCAR/miR-34a-5p is processed to miR-34a-5p in human ES cells to modulate target gene expression and inhibit cell proliferation. **(A)** Levels of miR-34a-5p were over 200-fold higher in A673 cells treated with nCAR/miR-34a-5p than control RNA or vehicle (*P* < 0.0001, one-way ANOVA), while there was no difference between control RNA and vehicle treatments. **(B,C)** Western blot analyses revealed a significant reduction of protein levels of SIRT-1, BCL-2, and CDK6 in A673 cells following the treatment with nCAR/miR-34a-5p, as compared to control RNA and vehicle (*P* < 0.0001, two-way ANOVA). **(D,E)** Bioengineered nCAR/miR-34a-5p showed a dose-dependent inhibition of human ES A673 cell and RD-ES cell proliferation (*P* < 0.001, two-way ANOVA), and **(F,G)** pharmacodynamic parameters were estimated by fitting the data into an inhibitory dose-response model with variable slope Y = Emin + (Emax − Emin)/[1 + 10^(LogEC50−X)^*HillSlope]. Values are mean ± SD of triplicate treatments. *P < 0.05; **P < 0.01; ***P < 0.001; ****P < 0.0001.

To delineate the pharmacological actions of bioengineered nCAR/miR-34a-5p in the modulation of human ES A673 cellular processes, we quantified the mature miR-34a-5p levels through selective stem-loop reverse transcription qPCR assay and determined the protein levels of miR-34a-5p target genes through immunoblot analysis. Interestingly, A673 cells treated with bioengineered nCAR/miR-34a-5p showed over 200-fold higher levels of mature miR-34a-5p than cells treated with control RNA or vehicle (*P* < 0.0001, one-way ANOVA, Figure 1A), while there was no significant difference between cells treated with control RNA and vehicle. These data demonstrated that bioengineered nCAR/miR-34a-5p was successfully transfected into human ES A673 cells and processed to mature miR-34a-5p.

As SIRT-1, BCL-2, and CDK6 are well-defined targets of miR-34a-5p and involved in cell apoptosis and cycle arrest (35–39), Western blot analyses were thus conducted to define the impact of bioengineered nCAR/miR-34a-5p on target gene expression. The results showed that nCAR/miR-34a-5p reduced the protein levels of SIRT-1, BCL-2 and CDK6 to much lower degrees than control RNA and vehicle treatment (*P* < 0.0001, two-way ANOVA, Figures 1B,C), supporting the utility of bioengineered nCAR/miR-34a-5p in the modulation of target gene expression.

To further evaluate the antiproliferative activity of bioengineered nCAR/miR-34a-5p, cell viability was determined using CCK-8 assay. The results showed that proliferation of ES A673 cells and RD-ES cells was significantly suppressed by nCAR/miR-34a-5p in a dose-dependent manner, as compared to control RNA (*P* < 0.001, two-way ANOVA; Figures 1D,E). The antiproliferative activity of bioengineered nCAR/miR-34a-5p was further manifested by the calculated EC50, Hill Slope, Top and Bottom values, compared to those for control RNA (Figures 1F,G). The lower EC50 and Bottom (greater inhibition) values revealed a greater effect of nCAR/miR-34a-5p on cell proliferation of A673 than that of RD-ES. Moreover, seeing from the higher tumorigenicity and typicality of A673, it was chosen for further assessments *in vitro* and in vivo rather than RD-ES. Together, these findings demonstrated that high-levels of mature miR-34a-5p was successfully released from bioengineered nCAR/miR-34a-5p and consequently exerted antiproliferative activity against human ES cells through down-regulation of target oncogene expression.

### Bioengineered nCAR/miR-34a-5p Induces Apoptosis in Ewing Sarcoma Cells

**Figure 2:**
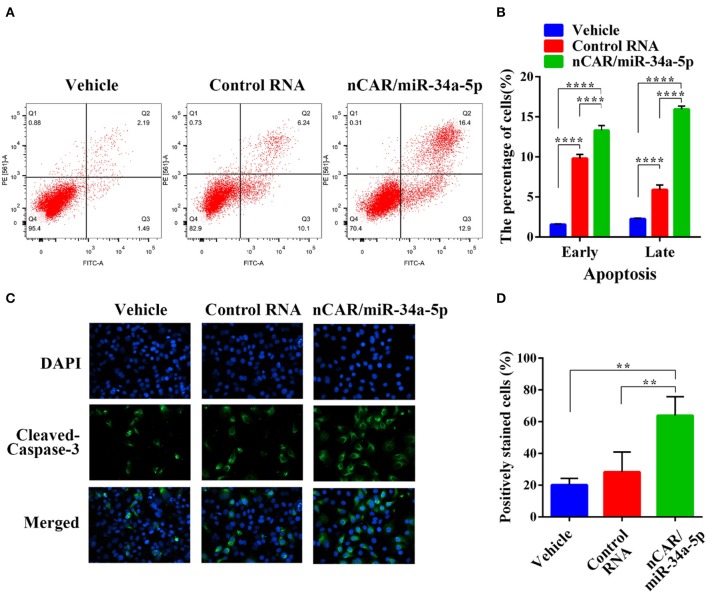
Bioengineered nCAR/miR-34a-5p induces apoptosis in ES cells. **(A)** The apoptotic profiles were evaluated by flow cytometric analysis after Annexin V-FITC/PI staining. **(B)** Comparison of the percentages of apoptotic cells with different treatments showed that bioengineered nCAR/miR-34a-5p largely induced apoptosis (*P* < 0.0001, two-way ANOVA). **(C–D)** Immunofluorescence assessment of apoptotic biomarker cleaved-casepase-3 in ES A673 cells further confirmed the effect of bioengineered nCAR/miR-34a-5p on apoptosis, which was quantitated as over 100% increase in the number of cleaved-caspase-3-positive cells treated with nCAR/miR-34a-5p, as compared to either control RNA or vehicle treatment (*P* < 0.01, one-way ANOVA). Values are mean ± SD of triplicate treatments. *P < 0.05; **P < 0.01; ***P < 0.001; ****P < 0.0001.

To evaluate the impact of nCAR/miR-34a-5p on apoptosis, flow cytometric analysis was conducted to examine the apoptotic profiles after Annexin V-FITC/PI staining of cells with different treatments (Figure 2A). The results indicated that the fractions of apoptotic cells treated with bioengineered nCAR/miR-34a-5p were much greater than control RNA or vehicle group (*P* < 0.0001, two-way ANOVA; Figure 2B). In particular, late apoptosis (170.3% increase) was altered to a greater degree than early apoptosis (35.5% increase) by nCAR/miR-34a-5p, compared with control RNA.

To further verify the action of bioengineered nCAR/miR-34a-5p on apoptosis, immunofluorescence analysis was performed to determine the apoptosis biomarker cleaved-caspase-3 (48). It was worth noting that bioengineered nCAR/miR-34a-5p treated group showed higher intensity of fluorescence signals in comparison with vehicle and control RNA group (Figure 2C). Cleaved-caspase-3-positive cells count data also demonstrated that normalized cleaved-caspase-3-positive cell number (percentage of cleaved-caspase-3-positive cells) of bioengineered nCAR/miR-34a-5p treated group was (63.87–28.31%)/28.31% = 125.6% higher than that of control RNA, and more than 2 times higher than that of vehicle (*P* < 0.01, one-way ANOVA; Figure 2D), while there was no significant difference between control RNA group and vehicle. Together, the results suggested that bioengineered nCAR/miR-34a-5p was active in promoting apoptosis of human ES A673 cells that may contribute to its antiproliferative activity (Figure 1D).

### Bioengineered nCAR/miR-34a-5p Causes a G2 Cell Cycle Arrest in Ewing Sarcoma Cells

**Figure 3:**
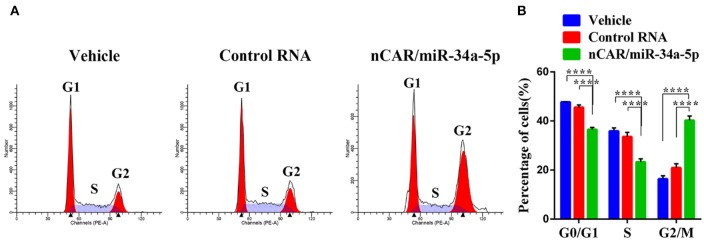
Bioengineered nCAR/miR-34a-5p causes a G2 cell cycle arrest in ES cells. **(A)** Cell cycle profiles were determined by flow cytometry analysis after the cells were stained with propidium iodide for DNA contents. **(B)** Quantitative measurements showed that nCAR/miR-34a-5p treatment led to a remarkable accumulation of ES A673 cells in G2 phase, while the numbers of ES A673 cells in G0/G1 and S phase were reduced accordingly (*P* < 0.0001, two-way ANOVA). Values are mean ± SD of triplicate treatments. *P < 0.05; **P < 0.01; ***P < 0.001; ****P < 0.0001.

To investigate how ES cell cycle profiles were influenced by bioengineered nCAR/miR-34a-5p, drug-treated A673 cells were subjected to cell cycle analysis by flow cytometry following PI-staining. Compared with the control RNA or vehicle, nCAR/miR-34a-5p remarkably altered the cell cycle profiles (Figure 3A). Specifically, nCAR/miR-34a-5p led to an accumulation of A673 cells in G2 phase, while the percentages of A673 cells in G0/G1 and S phases were reduced accordingly (*P* < 0.0001, two-way ANOVA; Figure 3B). These results demonstrated that the antiproliferative activity (Figure 1D) of bioengineered nCAR/miR-34a-5p was associated with the induction of G2 cell cycle arrest in ES cells.

### Bioengineered nCAR/miR-34a-5p Effectively Inhibits Tumor Growth in Subcutaneous Ewing Sarcoma Xenograft Mouse Models

**Figure 4:**
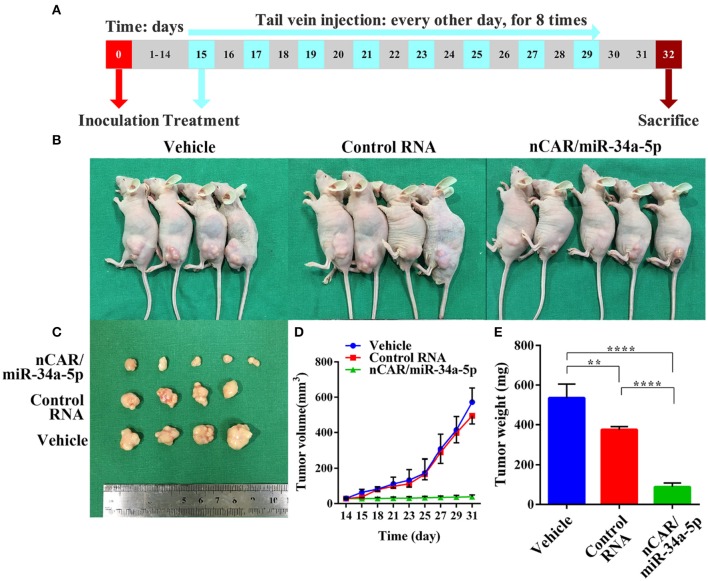
Bioengineered nCAR/miR-34a-5p effectively inhibits tumor growth in ES xenograft mouse models. **(A)** The scheme of therapy study *in vivo* following the establishment of ES xenograft mouse models. **(B,C)** Systemic administration of bioengineered nCAR/miR-34a-5p led to an obvious and large suppression of ES tumor progression than control RNA and vehicle treatment. **(D)** Comparison of xenograft tumor growth between different treatments. The nCAR/miR-34a-5p treated mice had a significant decrease in tumor volumes from day 21 after inoculation, compared to control RNA (*P* < 0.05, two-way ANOVA) and vehicle (*P* < 0.01, two-way ANOVA) treated mice. On day 31, tumor volumes in nCAR/miR-34a-5p treatment group were no more than 1/12 of that in control RNA group and 1/14 of that in vehicle (*P* < 0.0001, two-way ANOVA). **(E)** Comparison of the weights of xenograft tumor tissues (*P* < 0.0001, one-way ANOVA). Values are mean ± SD of triplicate treatments. *P < 0.05; **P < 0.01; ***P < 0.001; ****P < 0.0001.

To investigate the efficacy of nCAR/miR-34a-5p in the control of ES tumor growth, xenograft tumor mouse models were established via subcutaneous inoculation of A673 cells (Figure 4A). Tumor-bearing mice were then randomized into three groups to receive intravenous administration of *in vivo*-jetPEI-formulated nCAR/miR-34a-5p, control RNA and vehicle, respectively. It was obvious that nCAR/miR-34a-5p treatment led to a significant suppression of the outgrowth of viable tumors, compared to the control RNA and vehicle treatments (Figure 4B). The consistent tendency was also indicated by visual inspection of *ex vivo* tumor sizes and quantitative measurements of tumor weights and volumes (Figures 4C–E). From day 21 after inoculation, the mice treated with nCAR/miR-34a-5p had a remarkable decline of the outgrowth of tumor volumes compared with control RNA (*P* < 0.05, two-way ANOVA; Figure 4D) and vehicle treatments (*P* < 0.01, two-way ANOVA; Figure 4D). At the endpoint of study, the tumor volumes of mice with nCAR/miR-34a-5p treatment were smaller than 1/12 of that in control group and 1/14 of that in vehicle group (*P* < 0.0001, two-way ANOVA; Figure 4D).

**Figure 5:**
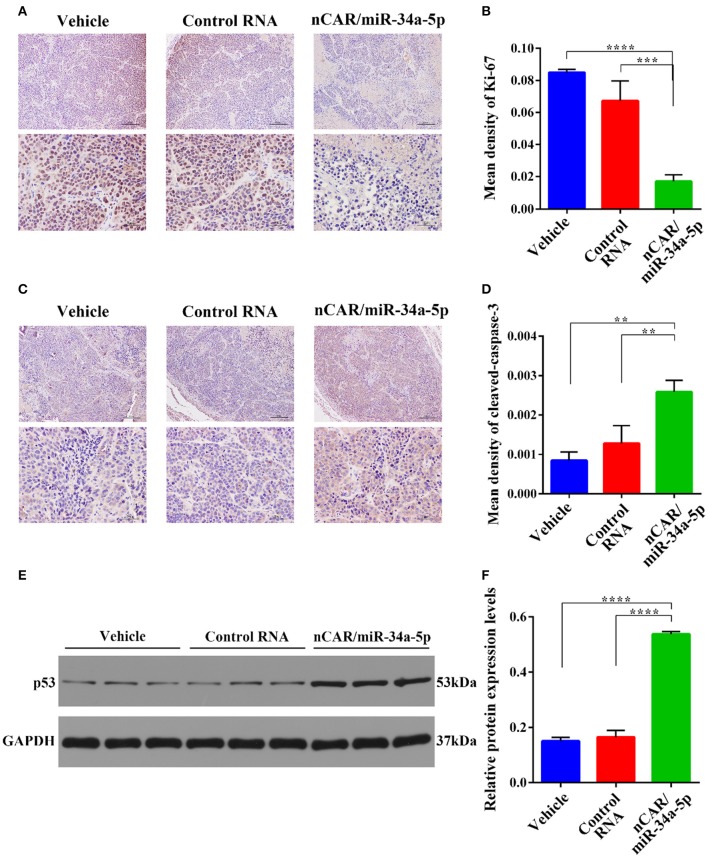
Immunohistochemistry and Western blot analyses of xenograft tumor tissues from different treatment groups. **(A)** The proliferative biomarker Ki-67. Upper scale bar: 200 μm; lower scale bar: 50 μm. **(B)** Quantitative comparison of Ki-67 levels revealed that bioengineered nCAR/miR-34a-5p greatly inhibited cell proliferation in xenograft tumors, as compared to control RNA (*P* < 0.001, one-way ANOVA) or vehicle treatment (*P* < 0.0001, one-way ANOVA). **(C)** The apoptotic biomarker cleaved-caspase-3. Upper scale bar: 200 μm; lower scale bar: 50 μm. **(D)** Quantitative comparison of cleaved-caspase-3 levels demonstrated that nCAR/miR-34a-5p treatment largely induced apoptosis in xenograft tumors, compared with either control RNA or vehicle treatment (*P* < 0.01, one-way ANOVA). **(E,F)** Western blot analyses revealed a significant increase of p53 protein level in tumor tissues following the treatment with nCAR/miR-34a-5p, as compared to control RNA and vehicle (*P* < 0.0001, one-way ANOVA). Values are mean ± SD of triplicate treatments. *P < 0.05; **P < 0.01; ***P < 0.001; ****P < 0.0001.

Dissected tumor tissues were further processed for immunohistochemistry analyses. The levels of the well-defined proliferation biomarker Ki-67 in nucleus (49) (Figure 5A), and the apoptosis biomarker cleaved-caspase-3 within cytoplasm (48) (Figure 5C) were examined to evaluate tumoral cell proliferative and apoptotic statuses, respectively. The data showed that Ki-67 contents in the tumor tissues from nCAR/miR-34a-5p-treated animals were significantly lower than those treated with control RNA (*P* < 0.001, one-way ANOVA; Figures 5A,B) or vehicle (*P* < 0.0001, one-way ANOVA; Figures 5A,B). By contrast, the levels of cleaved-caspase-3 in nCAR/miR-34a-5p-treated mouse tumors were significantly higher than control RNA (>2-fold, *P* < 0.01, one-way ANOVA; Figures 5C,D) or vehicle group (>3-fold, *P* < 0.01, one-way ANOVA; Figures 5C,D).

To further explore the signaling pathway involved in the control of ES tumor growth by nCAR/miR-34a-5p, western blot analyses of p53, an upstream protein in the positive feedback loop in p53, miR-34a, and SIRT-1 (35), were performed on tumor tissues after different treatments. The results revealed an elevation of p53 expression in tumors treated with nCAR/miR-34a-5p compared with vehicle or with control RNA (*P* < 0.0001, one-way ANOVA; Figures 5E,F).

Together, these findings indicated that bioengineered nCAR/miR-34a-5p was effective to inhibit ES tumor progression in xenograft mouse models *in vivo*, which was associated with lower degrees of tumoral cell proliferation and higher levels of apoptosis.

### Therapeutic Doses of nCAR/miR-34a-5p Is Well Tolerated in Mice

**Figure 6:**
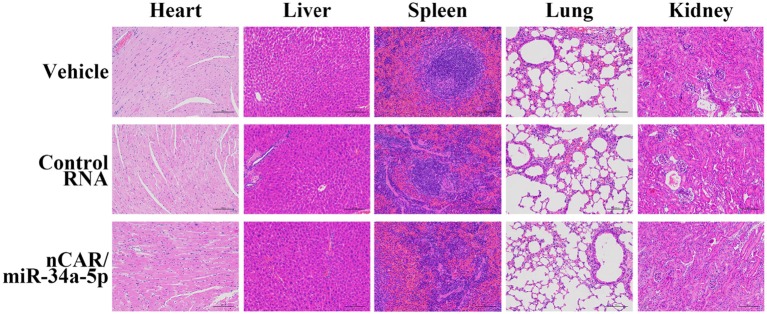
Bioengineered nCAR/miR-34a-5p does not cause any toxicity to major organs in mice *in vivo*. Histological analysis of excised organs at the end of therapy study revealed no obvious hydropic damage or necrotic lesions by bioengineered nCAR/miR-34a-5p treatments. Scar bar: 100 μm.

**Figure 7:**
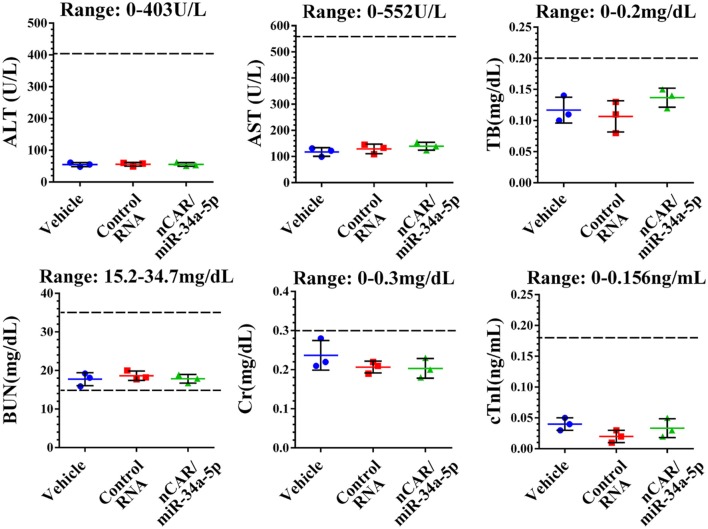
Bioengineered nCAR/miR-34a-5p does not cause any hepatic, renal or cardiac toxicity in mouse models *in vivo*. The blood chemistry profiles, including ALT, AST, TB, BUN, Cr and cTnI, were all within the normal ranges, indicating a good biocompatibility of bioengineered nCAR/miR-34a-5p in mouse models *in vivo*.

During the treatment, all mice did not show any signs of stress such as hunched posture or labored movement. While some mice in each experimental group showed a slight and acceptable loss of body weights at the endpoint of study, the majority of mice exhibited a normal body weight range. Most importantly, there were no significant differences in body weights between any treatment groups. Moreover, histological analyses of major organs in all treatment groups, including heart, liver, spleen, lung, and kidney, excised at the end of therapy study showed no obvious hydropic damage or necrotic lesions (Figure 6). Additionally, serum samples were collected and subjected to blood chemistry profile analysis, including alanine aminotransferase (ALT), aspartate aminotransferase (AST), total bilirubin (TB), blood urea nitrogen (BUN), creatinine (Cr) and cardiac troponin-I (cTnI), for the evaluation of possible liver, kidney, and heart toxicities. The data showed that none of these blood biomarkers was significantly altered by nCAR/miR-34a-5p therapy, compared to control RNA or vehicle treatments, and all markers were within normal ranges (Figure 7), indicating the absence of hepatic, renal and myocardial toxicity. The findings indicated that systemic administration of bioengineered nCAR/miR-34a-5p was well tolerated and relatively safe in xenograft ES mouse models.

## Discussion

Evidence is emerging that non-coding miRNAs play important roles in the control of various types of cancer diseases including initiation, progression, and metastasis (1, 2, 9). In the past decades, there are also increasing numbers of studies demonstrating the potential of RNA therapeutics, with the understanding of aberrant expression of miRNAs in carcinoma cells that contributes to the development and progression of cancer diseases (12–15). As an example, miR-34a replacement therapy using MRX34, a synthetic miR-34a mimic encapsulated in liposome, exhibits effective anticancer activities for the treatment of unresectable primary liver cancer in a Phase I clinical trial (50). Unfortunately, the trial was terminated due to serious adverse events, despite that the exact causes remained elusive (50).

Currently, chemically-engineered miRNA mimics have been predominately employed for miRNA research and development because of an easy accessibility. Nevertheless, due to the excessive artificial modifications, there are likely unknown alterations in the structures, activities and toxicities between synthetic and natural RNA molecules, which have not been fully explored yet (41–43). Very recently, we have successfully established a novel ncRNA bioengineering technology using a tRNA/pre-miRNA-based carrier to achieve high-yield and large-scale production of highly-structured, stable miRNA agents with minimal post-transcriptional modifications (43, 44). Unlike chemically-engineered miRNAs, the bioengineered miRNA prodrug is naturally synthesized and folded within the live cells which permits better capture of the properties of natural RNA molecules *in vitro* and *in vivo* (43, 44). Because of the good biocompatibility, bioengineered miRNA prodrugs should be more suitable for the assessment of RNA therapeutics beyond conventional chemo-engineered miRNA mimics (44–47).

Being the second most common type of primary bone malignancy, ES encounters the dilemma of unimproved survival rate with a lack of effective treatments (25–27). With a better understanding of miRNA functions in ES, a variety of miRNAs such as miR-30a-5p, miR-125b, and miR-145 have been identified to be down-regulated in ES; and thus, restoration of a tumor suppressive miRNA is effective to modulate the initiation and progression of ES (51–53). MiR-34a is a tumor suppressive miRNA modifying multiple downstream targets which are implicated in tumorigenesis and cancer progression, such as MYC, NOTCH1, CDK4/6, BCL-2, c-MET, and CD44 (54, 55), as well as SIRT1 (35, 36). Given the findings on a significantly lower miR-34a level in ES (32–34), along with the fact that a lower miR-34a expression level is correlated with a notably poor prognosis in ES patients (34), we recognize them as evidences of “anti-miR-34a” effect on ES tumor growth. Thus, this study aimed to employ the exogenous introduction of novel bioengineered miR-34a-5p prodrug as a strategy for the treatment of ES. Our data demonstrated high levels of mature miR-34a-5p was selectively released from bioengineered miR-34a-5p prodrug in human ES A673 cells. Consequently, bioengineered miR-34a-5p prodrug exerted potent antiproliferative activity against ES with an enhancement of apoptosis and induction of G2 cell cycle arrest, attributable to a set of proteins (apoptosis-associated SIRT-1, BCL-2 and cell cycle arrest-associated CDK6) down-regulated by miR-34a-5p prodrug that were assembled into critical tumor regulatory pathways. The remarkable suppression of ES xenograft tumor growth by miR-34a-5p prodrug, revealed in current study, was associated with a lower degree of tumoral cell proliferation and greater extent of apoptosis, as well as a relatively increased level of p53 expression, providing a good molecular explanation for the effectiveness of miR-34a-5p prodrug in xenograft mouse models.

Moreover, miR-34a-5p prodrug therapy did not cause significant body weight loss in the mice. Blood chemistry profile showed that the levels of ALT, AST, TB, BUN, Cr, and cTnI were all within the normal ranges. Cell necrosis or inflammatory infiltrate was not observed in histological analyses of major organs. It is concluded that therapeutic doses of bioengineered miR-34a-5p prodrug was well-tolerated in tumor-bearing mouse models *in vivo*. The favorable biocompatibility and low cytotoxicity of bioengineered miR-34a-5p prodrug *in vivo* suggest possible clinical applications, whereas more extensive safety studies are warranted.

It is well-known that p53 acts as key protein evoking cell activities including apoptosis, cell cycle arrest, DNA damage repair and senescence through modulation of its pathway (56). Increasing studies showed that the functionally intact p53 pathway is retained in the majority of ES cases (57, 58). As an important regulator in p53 pathway, miR-34a is able to stimulate endogenous p53 activity in a positive feedback-loop through inhibiting the expression of target gene SIRT-1 and thus leads to cell apoptosis (28, 29, 36). Our results revealed a remarkable down-regulation of SIRT-1 in ES cells and demonstrated obvious tendency of apoptosis both *in vitro* and *in vivo*, indicating that exogenous restoration of miR-34a-5p into ES cells could promote p53 activation and succumb ES to a single hit. In addition, EWS-FLI1, the most common fusion gene of ES, has been proven to regulate expression profiles of numerous miRNAs in ES, including miR-22, miR-30a-5p, and most notably miR-145 and let-7a (51–53, 56). Moreover, miR-34a has been identified as viable predictor of risk for ES progression and survival, along with other miRNAs (32–34). Nevertheless, the broad implication of miR-34a in EWS-FLI1 remains unknown and may be further investigated in the future.

## Conclusion

In summary, we investigated the exogenous introduction of bioengineered miR-34a-5p prodrug as a new strategy for the control of ES in human ES cells and xenograft mouse models. Our studies demonstrated that bioengineered nCAR/miR-34a-5p, a novel miRNA prodrug produced by our newly established ncRNA bioengineering technology, was selectively processed to high level of mature miR-34a-5p in ES cells and subsequently, suppressed ES cell proliferation. The pharmacological effects of miR-34a-5p was attributable to the enhancement of apoptosis and induction of G2 cell cycle arrest through downregulation of SIRT-1, BCL-2 and CDK6 protein levels. In addition, systemic administration of miR-34a-5p prodrug dramatically suppressed the ES xenograft tumor growth *in vivo* while well-tolerated in mice. The antitumor effect of miR-34a-5p prodrug was associated with a lower degree of tumoral cell proliferation and greater extent of apoptosis. Overall, the optimal outcomes of antitumor activity and safety of bioengineered miR-34a-5p prodrug support the development of miRNA therapeutics using novel biocompatible bioengineered miRNA molecules.

## Data Availability Statement

All datasets generated for this study are included in the article.

## Ethics Statement

The animal study was reviewed and approved by Zhongnan Hospital of Wuhan University.

## Author Contributions

A-XY and CJ designed the research. D-FL and YY performed the study, conducted the experiments, and analyzed the data. M-JT, XH, Y-ZL, W-RY, P-CL, and YZ performed parts of the experiments. D-FL and YY wrote the manuscript. ZC, A-MY, and A-XY oversaw the study and revised the manuscript. All authors read and approved the final manuscript.

### Conflict of Interest

The authors declare that the research was conducted in the absence of any commercial or financial relationships that could be construed as a potential conflict of interest.

## Abbreviations

ES: Ewing Sarcoma
miR-34a: microRNA-34a
ncRNA: non-coding RNA
nCAR: non-coding RNA carrier
EWSR1-FLI1: Ewing Sarcoma breakpoint region 1 protein-Friend leukemia integration 1 transcription factor
SIRT-1: silent information regulator-1
CDK6: cyclin-dependent kinase 6
BCL-2: B-cell lymphoma-2
c-MET: hepatocyte growth factor receptor
CD44: cell surface glycoprotein
ALT: alanine aminotransferase
AST: aspartate aminotransferase
TB: total bilirubin
BUN: urea nitrogen
Cr: creatinine
cTnI: cardiac troponin-I
CCK-8: Cell Counting Kit-8
RT-qPCR: reverse transcription quantitative real-time polymerase chain reaction
HPLC: high-performance liquid chromatography
i.p.: intraperitoneal
IHC: immunohistochemical staining.
